# Root infection by the nematode *Meloidogyne incognita* modulates leaf antiherbivore defenses and plant resistance to *Spodoptera exigua*

**DOI:** 10.1093/jxb/erab370

**Published:** 2021-09-21

**Authors:** Crispus M Mbaluto, Fredd Vergara, Nicole M van Dam, Ainhoa Martínez-Medina

**Affiliations:** 1 Molecular Interaction Ecology, German Center for Integrative Biodiversity Research (iDiv) Halle-Jena-Leipzig; PuschStraße 4, 04103, Leipzig, Germany; 2 Institute of Biodiversity, Friedrich-Schiller-Universität-Jena; DornburgerStraße 159, 07743 Jena, Germany; 3 Plant-Microorganism Interaction, Institute of Natural Resources and Agrobiology of Salamanca (IRNASA-CSIC), Cordel de Merinas, 40, 37008, Salamanca, Spain; 4 The James Hutton Institute, UK

**Keywords:** Aboveground–belowground interactions, *Meloidogyne incognita*, phytohormones, plant-mediated interactions, untargeted metabolomics, root-knot nematode, *Spodoptera exigua*, systemic induced responses

## Abstract

Studies on plant-mediated interactions between root parasitic nematodes and aboveground herbivores are rapidly increasing. However, outcomes for the interacting organisms vary, and the mechanisms involved remain ambiguous. We hypothesized that the impact of root infection by the root-knot nematode *Meloidogyne incognita* on the performance of the aboveground caterpillar *Spodoptera exigua* is modulated by the nematode’s infection cycle. We challenged root-knot nematode-infected tomato plants with caterpillars when the nematode’s infection cycle was at the invasion, galling, and reproduction stages. We found that *M. incognita* root infection enhanced *S. exigua* performance during the galling stage, while it did not affect the caterpillar’s performance at the invasion and reproduction stages. Molecular and chemical analyses performed at the different stages of the nematode infection cycle revealed that *M. incognita* root infection systemically affected the jasmonic acid-, salicylic acid-, and abscisic acid-related responses, as well as the changes in the leaf metabolome triggered during *S. exigua* feeding. The *M. incognita*-induced leaf responses varied over the nematode’s root infection cycle. These findings suggest that specific leaf responses triggered systemically by the nematode at its different life-cycle stages underlie the differential impact of *M. incognita* on plant resistance against the caterpillar *S. exigua*.

## Teaser

The impact of root infection by the nematode *Meloidogyne incognita* on leaf anti-herbivore defenses and plant resistance to the caterpillar *Spodoptera exigua* is modulated by the nematode infection cycle.

## Introduction

In natural and agricultural ecosystems, plants are constantly interacting with a multitude of organisms that attack the roots and the shoots. To counteract the attack by enemies, plants possess a sophisticated immune system that recognizes non-self molecules or signals from their own injured cells (Duran-Flores and Heil, 2016). They respond by activating an immune response against the invader (Pieterse *et al.*, 2012). Plant hormones such as jasmonic acid (JA) and its derivatives (jasmonates), salicylic acid (SA), ethylene, and abscisic acid (ABA) are central players in the regulation of the plant immune network (Pieterse *et al.*, 2009). After the attack by enemies, induced defenses are usually expressed not only at the damaged tissue, but also systemically in non-attacked plant parts (Biere and Goverse, 2016). The systemic response enables the plant to protect undamaged tissues and can affect the performance of other organisms feeding on the same plant (van Dam *et al.*, 2005; Hol *et al.*, 2013; Hoysted *et al.*, 2017; Arce *et al.*, 2017). Besides changes in plant immunity, plant interactions with pathogens and herbivorous insects can affect the plant’s nutritional status and nutrient allocation patterns. Such changes in primary plant metabolism can have profound consequences on the performance of herbivores feeding on the same plant (Berenbaum, 1995). As a consequence, plants are essential mediators of interactions between organisms that rarely come into direct physical contact with one another (Soler *et al.*, 2013).

Indeed, previous studies demonstrated that belowground (BG) organisms that closely associate with plant roots influence primary and secondary metabolism in aboveground (AG) plant parts, affecting the growth and development of herbivores feeding on AG tissues (Erb *et al.*, 2009; Kumar *et al.*, 2016; Hoysted *et al.*, 2017; Arce *et al.*, 2017; van Dam *et al.*, 2018). These studies report both positive and negative effects of BG organisms on the performance of AG herbivores, depending on the study system. For instance, root infection by *Meloidogyne hapla* on *Brassica nigra* plants resulted in stronger attraction of *Brevicoryne brassicae* aphids compared with non-infected plants (van Dam *et al.*, 2018). By contrast, root infection by *Meloidogyne incognita* reduced both oviposition and performance of the leaf miner *Tuta absoluta* in tomato plants (Arce *et al.*, 2017). An increasing number of studies aim to disentangle the mechanisms underpinning the effects of BG herbivores on herbivorous insects feeding on AG tissues (Bezemer *et al.*, 2005; Soler *et al.*, 2007; Kaplan *et al.*, 2009; Hoysted *et al.*, 2017; Arce *et al.*, 2017; van Dam *et al.*, 2018). Most of these studies focus on root-chewing herbivores, whereas studies of AG–BG interactions involving plant–parasitic nematodes are relatively rare.

Root-knot nematodes (RKNs) are tiny multicellular organisms that parasitize the root systems of thousands of plants. They reprogram plant processes in roots to ensure a continuous supply of resources (Gheysen and Mitchum, 2011). The RKN infection cycle has different stages, including the invasion of the host roots, followed by establishment in the root tissues, and reproduction (Mbaluto *et al.*, 2020). Once the infective juveniles hatch, they pierce and penetrate the roots at the zone of elongation. They move intercellularly downwards to the root tip where they enter into the vascular cylinder, then turn around and move intercellularly upwards until they reach the differentiation zone where they settle and induce the formation of feeding sites (Escobar *et al.*, 2015). They select six to eight vascular cells that they pierce with their stylet by means of which they inject pharyngeal gland secretions. These secretions cause the re-differentiation of cells into multinucleate and hypertrophied feeding cells (i.e. the feeding sites), which are commonly called giant cells (Caillaud *et al.*, 2008; Bozbuga *et al.*, 2018). This entire process results in the formation of visible structures called root-knots or galls (Escobar *et al.*, 2015). We recently demonstrated that root responses triggered by the RKN *M. incognita* infection differ significantly through the nematode infection cycle. Our results show that *M. incognita* root infection increased the endogenous concentrations of JA, SA, and ABA in tomato roots specifically when it reaches the reproduction stage (Mbaluto *et al.*, 2020). Along the same lines, plant genes associated with signal transduction, secondary metabolism, and defense can be up-regulated specifically at the onset of the nematode infection (Hamamouch *et al.*, 2011). At later stages of root infection, genes encoding peroxidases, major intrinsic proteins, and glucose are repressed (Portillo *et al.*, 2013). These differences in nematode-induced responses are relevant because nematode-triggered root responses can also systemically affect induced responses in AG organs (Hamamouch *et al.*, 2011; Kyndt *et al.*, 2014). In Arabidopsis, for instance, *M. incognita* root infection triggers the expression of SA- and JA-related genes in roots, but suppresses them in shoots (Hamamouch *et al.*, 2011). Besides the modulation of AG defense responses, several studies show that some of the changes in primary plant metabolism triggered by RKNs are not restricted to the roots, but can also be expressed in AG tissues (Kyndt *et al.*, 2014). This systemic impact of parasitic root nematodes on defenses and primary metabolism has been associated with changes in the performance of herbivores feeding on AG plant parts (Hol *et al.*, 2013; Hoysted *et al.*, 2017; Arce *et al.*, 2017). However, the outcomes of the interaction between RKN and AG insect herbivores are variable, and positive, negative, as well as neutral effects have been reported (Kaplan *et al.*, 2009; Kafle *et al.*, 2017).

In this study, we explored the plant-mediated root-to-shoot interaction between the RKN *M. incognita* and the caterpillar *Spodoptera exigua* in tomato (*Solanum lycopersicum*). Using a combination of glasshouse bioassays and molecular and chemical analyses, we tested the hypothesis that the impact of root infection by *M. incognita* on AG defense responses and plant resistance to the AG insect herbivore *S. exigua* depends on the specific stage of the nematode’s infection cycle. We found that *M. incognita* enhanced the performance of *S. exigua* specifically at the galling stage. Our results further demonstrate that *M. incognita* root infection affected JA-, SA-, and ABA-related responses in the leaves as well as the metabolic response triggered by *S. exigua* feeding. Moreover, this effect was dependent on the specific nematode infection cycle stage at which the caterpillar was feeding on the leaves. Collectively, our study demonstrates that the impact of root infection by *M. incognita* on the plant’s interaction with the AG herbivorous insect *S. exigua* is dependent on the nematode’s infection cycle.

## Materials and methods

### Plant material and growing conditions

Tomato (*Solanum lycopersicum* cv. Moneymaker) was used as a model plant in all experiments. We obtained tomato seeds from Intratuin BV (Woerden, The Netherlands). Seeds were surface-sterilized by immersion in 10% sodium hypochlorite solution for 4 min. Subsequently, the seeds were rinsed four times with sterile water. The sterilized seeds were placed on tap water-moistened glass beads and allowed to germinate at 27 °C in the dark for 3 d, followed by 4 d in the light. When the seedlings were 7 d old, they were transplanted into a 1:1 sand: soil mixture in 11 cm×11 cm×12 cm pots. Seedlings were grown in a glasshouse for three more weeks, under 16 h light (26±3 °C) and 8 h dark (23±3 °C), according to Rodriguez-Saona *et al.* (2010). The plants were watered as required and supplemented with half-strength Hoagland solution (Hoagland and Arnon, 1938) weekly. Altogether, the plants were 4 weeks old at the beginning of experiments.

### Belowground and aboveground herbivores

The RKN *M. incognita* was used as the BG herbivore. Initial *M. incognita* eggs were kindly provided by Dr Adriaan Verhage (Rijk Zwaan, De Lier, The Netherlands) and used to maintain a glasshouse stock colony on *S. lycopersicum*. The inoculum was started from a single egg mass, and when the infected plants were approximately 8 weeks old, eggs were harvested and used for experiments (Martínez-Medina *et al.*, 2017). The generalist leaf chewer *S. exigua* was used as the AG herbivore. *Spodoptera exigua* eggs were purchased from Entocare CV Biologische Gewasbescherming (Wageningen, The Netherlands). The eggs were incubated and hatched, and the larvae were reared on an artificial diet according to Hoffman *et al.* (1967). The artificial diet consisted of the following ingredients (per 500 ml): 80 g cornflour, 25 g yeast extract, 25 g wheat germ, 1 g ascorbic acid, 0.8 g methyl-4-hydroxybenzoate, 0.05 g streptomycin, 8 g agar, and 500 ml Milli-pure water. To prepare the diet, we dissolved agar in water, then poured the dissovled agar into a blender and added all the other ingredients. After blending, the mixture was dispensed into clean and sterile plates and store at 4 °C. The *S. exigua* colony was maintained in a growth chamber (CLF PlantClimatic, CLF PlantClimatics GmbH, Wertingen, Germany), set at 25 °C, 45% relative humidity with a 12 h photoperiod cycle.

### Nematode infection and insect herbivore infestation

Plants were infected with herbivores when 4 weeks old. The plants assigned for *M. incognita* infection were inoculated with approximately 3000 *M. incognita* eggs per plant suspended in tap water. The inoculation was performed by injecting 1 ml of an egg suspension (3000 egg ml^−1^) into the soil close to the roots, according to Martínez-Medina *et al.* (2017). Plants that were not assigned for *M. incognita* inoculation were mock-inoculated with 1 ml^−1^ of water. We set three study time points: 5, 15, and 30 days post-inoculation (dpi), coinciding with the following stages of *M. incognita* root infection cycle: invasion (5 dpi), galling (15 dpi), and reproduction (30 dpi). At each time point, we infested the plants assigned for AG herbivory with four first-instar *S. exigua* larvae (for the assessment of AG herbivore performance), or one second-instar *S. exigua* larva (for molecular biology, chemical analyses, and elemental carbon and nitrogen analysis). The identification of the specific instars of *S. exigua* larvae was based on visual inspection.

### Bioassay for the assessment of *Spodoptera exigua* performance

To assess the performance of *S. exigua* larvae when feeding on tomato plants challenged or not challenged with *M. incognita*, we conducted a bioassay including the study time points as described above. We established two treatments, i.e. plants that were challenged aboveground with *S. exigua* alone and plants that were challenged belowground with *M. incognita* and aboveground with *S. exigua*. We used four first-instar *S. exigua* larvae. The larvae were placed on the adaxial surface of the third fully expanded leaf. On the leaf, the larvae were confined to a 7-cm (diameter) round clip cage (Bandoly and Steppuhn, 2016; Mbaluto *et al.*, 2020). We allowed the larvae to feed on the leaves of plants challenged with *M. incognita* at the invasion (5 dpi), galling (15 dpi), or reproduction (30 dpi) stage, or on plants not infected with *M. incognita* until they reached the pupa stage. A total of 15 biological replicates were established per treatment. Larvae were first allowed to feed on the plant for 6 d without disturbance. After that, at 2 d intervals, the larvae were removed and their weight was recorded. Larvae were returned to the same plant, on one leaf above the one they were previously feeding on, to ensure that larvae had enough food during the entire bioassay. This process was repeated throughout until all surviving larvae either reached the pupa stage or died. The pupae were then collected and monitored until they hatched into moths. During the bioassay, we recorded data on larval weight, pupal weight, sex determination from the pupae, and duration of the pupation process until hatching under a 25 °C, 12 h photoperiod, and 45% relative humidity regime. We also counted the number of root galls or root knots formed by *M. incognita* at the galling and reproduction stages by visual inspection (Supplementary Fig. S1).

### Bioassay for the assessment of tomato defensive and nutritional status

To assess the impact of *M. incognita* root infection and *S. exigua* caterpillar feeding on tomato leaf defenses and elemental carbon (C) and nitrogen (N) content, we conducted a further bioassay including the study time points after *M. incognita* inoculation (i.e. 5, 15, and 30 dpi) as described above. We used a single second-instar *S. exigua* caterpillar. On each plant, the *S. exigua* caterpillar was contained on the adaxial surface of the third fully expanded leaf. The *S. exigua* caterpillars were contained on the leaf using a 7-cm (diameter) clip cage, as mentioned above. In plants without leaf herbivory, an empty clip cage was set on a similar leaf as on plants with leaf herbivory. A total of 10 biological replicates were established per treatment. Caterpillars were allowed to feed on plants challenged with *M. incognita* at the invasion (5 dpi), galling (15 dpi), or reproduction (30 dpi) stage, or control plants for 24 h. Afterward, the damaged leaf (local leaf) was harvested and stored at –80 °C for gene expression and chemical analyses. For the analysis of trypsin protease inhibitor activity, we allowed *S. exigua* larvae to feed on the plants for 48 h, according to Steppuhn and Baldwin (2007) and Bandoly *et al.* (2015). The leaves were all harvested in the morning between 10.00 and 10.30 h on the respective harvest days.

### Determination of phytohormone concentrations

Plant hormones were extracted and quantified according to Machado *et al.* (2013) and Mbaluto *et al.* (2020). In brief, we extracted samples with the solvent ethyl acetate containing 40 ng of internal standards for each phytohormone: D_6_-JA, D_6_-jasmonyl-L-isoleucine (D_6_-JA-Ile), D_6_-ABA, and D_6_-SA. The levels of the phytohormones were analysed using liquid chromatography (Bruker Advance UHPLC, Bruker Daltonik, Bremen, Germany) coupled to a mass spectrometer (Bruker Elite EvoQ Triple quadrupole) (LC/MS EVOQ), as described by Schäfer *et al.* (2016). The separation was achieved on a Zorbax Eclipse XDB-C18 column (4.6 mm×50 mm, 1.8 µm, 80 Å, Agilent Technologies, Santa Clara, CA, USA), according to Mbaluto *et al.* (2020). Data acquisition and processing were performed using the ‘MS data review’ software (Bruker MS Workstation, version 8.2). Phytohormone levels were calculated based on the peak area of the corresponding internal standard and the amount of fresh weight (FW) of the leaf material (ng^−1^ mg^−1^ FW).

### Quantitative polymerase chain reaction analysis

Total RNA was extracted from ~100 mg FW of ground leaf material, according to Oñate-Sánchez and Vicente-Carbajosa (2008). Both quantitative and qualitative quality checks were performed using a NanoPhotometer P330 (Implen, Munich, Germany) and by gel electrophoresis (1% agarose). Traces of DNA were removed by treating 5 µg of the extracted RNA with 2 U µl^−1^ of DNaseI (Thermo Fisher Scientific, Schwerte, Germany) following the manufacturer’s instructions. We checked the quality of the cleaned RNA as mentioned above. First-strand cDNA was synthesized from 1 µg DNase-free RNA by reverse transcription using 200 U µl^−1^ Revert Aid H-minus RT (Thermo Fisher Scientific Baltic UAB, Vilnius, Lithuania) following the manufacturer’s instructions. The amplification cycle conditions for cDNA synthesis were: 42 °C for 60 min, 50 °C for 15 min, and 70 °C for 15 min using a thermal cycler (Techne, Stone, UK). Real-time quantitative PCR reactions were performed and relative quantification of specific mRNA levels were obtained using the CFX 384 Real-Time PCR system (Bio-Rad Laboratories Inc., Singapore) with gene-specific primers described in Supplementary Table S1. Reverse transcription–quantitative PCR (RT-qPCR) cycle conditions were: 2 min at 50 °C, 2 min at 95 °C, 40 cycles of 15 s at 95 °C, and 60 s at 60 °C (Vos *et al.*, 2015). Melting curve analysis was done to verify amplification of each gene transcript. Three technical replicates of each sample were included in the RT-qPCR. The data obtained were normalized using the reference gene *SIEF X14449*, which encodes the tomato elongation factor 1α (Miranda *et al.*, 2013; Martínez-Medina *et al.*, 2017). The stability of *SIEF* was previously evaluated in leaf tissues and under the different experimental conditions (nematode and caterpillar challenge) analysed here (see data deposited at iDiv in ‘Data availability’). Normalized gene expression data were analysed by the method (Livak and Schmittgen, 2001).

### Trypsin protease inhibitor activity analysis

To evaluate the trypsin protease inhibitor (TPI) activity in tomato leaves, we extracted total protein from 20 mg freeze-dried leaf material. The leaf samples were harvested 48 h after *S. exigua* feeding, according to Bandoly *et al.* (2015). The extraction and quantification process was carried out according to the radial diffusion method described by van Dam *et al.* (2001) and Bandoly *et al.* (2015).

### Determination of elemental carbon and nitrogen concentrations

Freeze-dried leaf material (~10 mg) was used for the determination of the elemental carbon and nitrogen concentration as a percentage. The samples were weighed into tin bowls and carefully compressed into a circular pellet. The pellets were incinerated and the released gases detected by a thermal conductivity detector in an elemental analyser (Elementar Analysensysteme GmbH, Langenselbold, Germany), according to Moreno-Pedraza *et al.* (2019).

### Metabolite extraction and data processing

To analyse the changes in tomato leaf metabolome, we extracted metabolites from ~20 mg (dry weight) leaf material. The extraction, quantification, and data analysis of the metabolites was carried out as described by De Vos *et al.* (2012), Rogachev and Aharoni (2012), and Moreno-Pedraza *et al.* (2019) with some modifications. We extracted every sample twice and combined the supernatants. We transferred 200 µl of the combined extracts into a 2 ml HPLC vial and added 800 µl of the extraction buffer to obtain a 1:5 dilution for each sample. We further prepared a 1:50 dilution of each sample by transferring 100 µl from each of the 1:5 dilutions into a new 2 ml HPLC vial and added 900 µl of the extraction buffer. The 1:50 dilution allowed us to correctly detect and quantify the tomatine peak without exceeding the mass analyser detection limit. We separated and characterized compounds by injecting 1 µl of each extract from the two dilutions (1:5 and 1:50) into a UPLC instrument (Dionex 3000, Thermo Scientific). The chromatograph was equipped with a C18 column (Acclaim TM RSLC 120), 2.1 mm×150 mm external dimension, 2.2 µm particle size, and 120 Å pore size. The column was kept at 40 °C. The mobile phases (LC-MS grade solvents) were composed of solvent A (0.05% (v/v) aqueous formic acid) and solvent B (0.05% (v/v) formic acid in acetonitrile). The multi-step gradient for solvent B was; 0−1 min 5%, 1−4 min 28%, 4−10 min 36%, 10−12 min 95%, 12−14 min 95%, 14−16 min 5%, and 16−18 min 5%. The flow was 400 µl min^−1^. We detected compounds using a maXis impact HD MS-qToF (Bruker Daltonik). Data were acquired in positive mode with similar settings to Moreno-Pedraza *et al.* (2019). We processed the data with MS-Dial, according to Moreno-Pedraza *et al.* (2019) with slight modifications for feature detection, retention time correction, and feature alignment. The parameters were: mass accuracy: MS1 tolerance=0.01 Da, retention time–begin=0.7 min, retention time–end=10 min, mass range–begin=50 mass to charge ratio (*m*/*z*), mass range–end=1500 *m*/*z*; peak detection parameters: minimum peak height=1000 amplitude, mass slice width=0.1 Da, smoothing method=linear weighted moving average, smoothing level=3 scans, minimum peak width=5 scans; alignment parameters settings: retention time tolerance=0.05 min, MS1 tolerance=0.015 Da. We normalized the alignments against the total ion chromatogram. We exported the normalized data matrix containing all the alignments as a .txt file (spectra type=centroid). We predicted metabolites by interpreting mass spectral features and by comparison against mass spectra deposited in the Mass Bank of North America database.

### Statistical analysis

Datasets were analysed using R software v 3.6.1 (2019; R Development Core Team) unless indicated otherwise. For the performance datasets, we used one-way ANOVA for statistical computations and detected differences between groups using Student’s *t*-test (*P*≤0.05) and the χ ^2^ test for the sex ratio dataset. In cases of defense response datasets, we used two-way ANOVA linear models consisting of *M. incognita*, *S. exigua*, and their interaction as model explanatory factors. We detected differences between groups by Tukey’s honest significant difference (HSD) for multiple comparisons (*P*≤0.05). All datasets were tested for normality and homogeneity of variance via visual inspection using Q–Q plots. We used the interquartile range rule for removing outliers in the datasets.

## Results

### Root infection by *Meloidogyne incognita* enhances *Spodoptera exigua* performance during the nematode galling stage

**Fig. 1.**
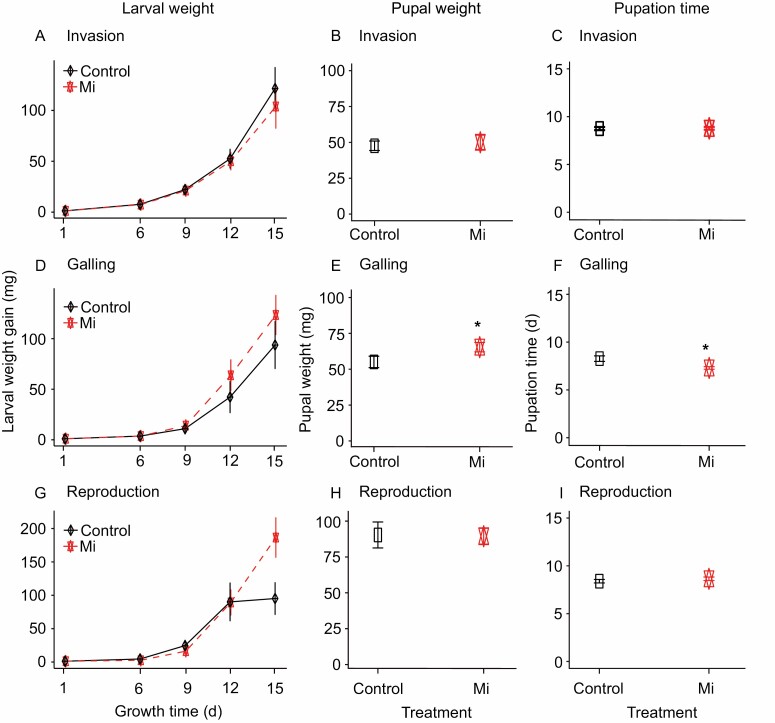
Impact of root infection by *Meloidogyne incognita* on the performance of *Spodoptera exigua*. *Spodoptera exigua* larval weight gain (A, D, G), pupal weight (B, E, H) and pupation time (C, F, I) were measured in *S. exigua* larvae and pupae collected from leaves of control plants, and from leaves of plants infected in roots with *Meloidogyne incognita* (Mi) at the invasion (A, B, C), galling (D, E, F), and reproduction (G, H, I) stages. Data are the mean ±standard error (*n*=15). Asterisks indicate significant differences between treatments, inferred by Student’s *t*-test at *P* ≤ 0.05.

We first tested the effect of *M. incognita* root infection cycle stages, including invasion (5 dpi), galling (15 dpi), and reproduction (30 dpi), on the performance of the aboveground chewing herbivore *S. exigua*. We found that in *M. incognita*-infected plants at the invasion (5 dpi) and reproduction (30 dpi) stages, *S. exigua* larval and pupal weight gain was similar to that observed in control plants (Fig. 1A, B, G, H; Supplementary Table S2). Only at the reproduction stage (30 dpi) we observed a higher weight gain (about 96%) in larvae after 15 d of feeding on *M. incognita*-infected plants compared with control plants (Fig. 1G). Moreover, neither the time of pupation nor the sex ratios of the emerging moths were significantly (*P*>0.05) affected by *M. incognita* root infection at invasion (5 dpi) and reproduction (30 dpi) stages (Fig. 1C, I; Table 1; Supplementary Table S2). Altogether these observations indicate that *M. incognita* root infection at the invasion and reproduction stages did not affect the performance of *S. exigua*.

**Table 1.** Chi-square test for the equality of *Spodoptera exigua* moth sex ratio

Treatment	Male	Female	Total per infection stage	χ^2^	*P*
Invasion stage					
Control	6	3	18	3.7387	0.053
Mi	2	7			
Galling stage					
Control	7	5	30	4.0594	**0.044**
Mi	4	14			
Reproduction stage					
Control	4	5	17	1.6721	0.196
Mi	6	2			
GLM ANOVA results^*a*^					
Time				1.7364	0.420
Mi				2.8735	0.090
Time×Mi				6.5967	**0.037**

Sex ratios were determined from *Spodoptera exigua* pupae collected from tomato plants without root infection (Control) and infected with *Meloidogyne incognita* (Mi). *Spodoptera exigua* infestation on Mi plants was performed either at the nematode’s invasion, galling, or reproduction stages. Data are the numbers of sex ratios counted per treatment and were analysed using one-way ANOVA. The differences between the treatments were detected by chi-square test at *P*≤0.05. Statistically significant effects are indicated in bold.

^*a*^ GLM, generalized linear model; time, the nematode infection cycle stages (invasion, galling, reproduction); Mi, *Meloidogyne incognita*.

Unlike at the invasion and reproduction stages, at the galling stage (15 dpi), *M. incognita* affected the *S. exigua* performance. At this specific stage, we observed that pupae collected from *M. incognita*-infected plants had a greater weight (Fig. 1E; Supplementary Table S2) and a shorter pupation period (Fig. 1F; Supplementary Table S2) compared with pupae collected from control plants. Moreover, we found a significantly (*P*=0.044) higher proportion of female *S. exigua* moths emerging on *M. incognita*-infected plants compared with controls (Table 1). In addition, our data showed an increase, albeit not statistically significant, in the larval weight of *S. exigua* that fed on plants infected with *M. incognita* compared with control plants (Fig. 1D; Supplementary Table S2). Overall, these results support our hypothesis that the impact of *M. incognita* root infection on AG feeding *S. exigua* is dependent on the nematode’s root infection cycle. In our study, root infection by *M. incognita* enhanced the performance of *S. exigua*, specifically during the galling stage (15 dpi).

### Root infection by *Meloidogyne incognita* alters the phytohormone-related leaf responses triggered by *Spodoptera exigua* feeding

**Fig. 2.**
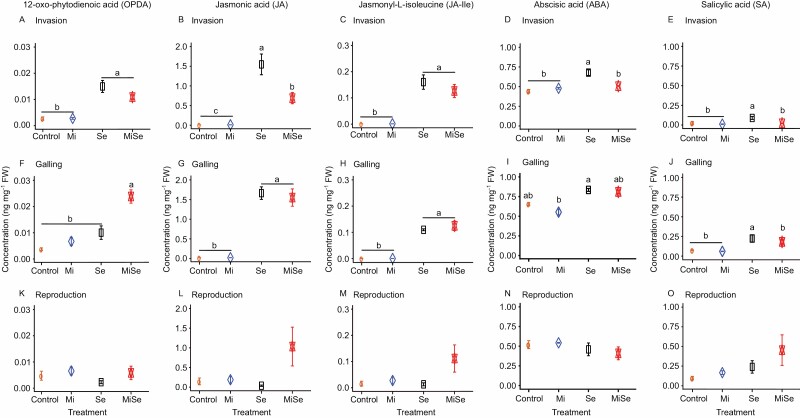
Concentrations of phytohormones in tomato leaves upon below- and aboveground herbivory. Concentrations of 12-oxo-phytodienoic acid (OPDA) (A, F, K), jasmonic acid (JA) (B, G, L), and jasmonyl-L-isoleucine (JA-Ile) (C, H, M), abscisic acid (ABA) (D, I, N), and salicylic acid (SA) (E, J, O) were measured in leaves of tomato plants without herbivores (Control), infected with *Meloidogyne incognita* (Mi) or *Spodoptera exigua* (Se) alone, or co-infected with both herbivores (MiSe). In co-infected plants, infestation with *S. exigua* was performed at the nematode invasion (A–E), galling (F–J), or reproduction (K–O) stage. Samples were taken 24 h after *S. exigua* feeding. Data are the mean ±standard error (*n*=5). Different letters indicate significant differences between treatments, determined by Tukey’s HSD test for multiple comparisons after two-way ANOVA at *P*≤0.05.

**Fig. 3.**
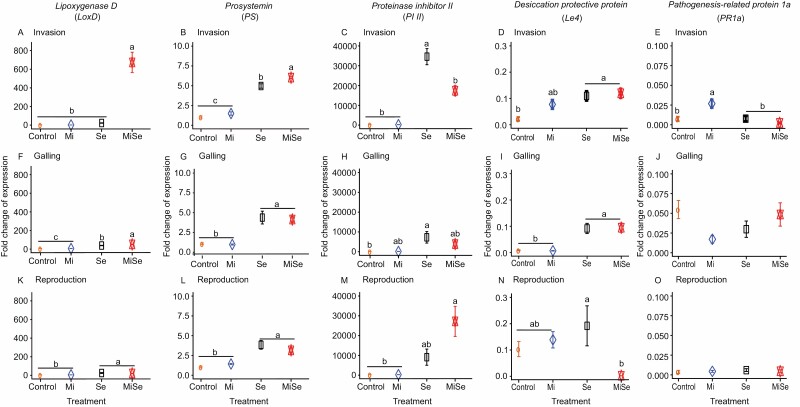
Expression patterns of marker genes in hormone pathways in tomato leaves upon below- and aboveground herbivory. Relative expression of *Lipoxygenase D* (*LoxD*) (A, F, K), *Prosystemin* (*PS*) (B, G, L), and *Proteinase inhibitor II* (*PI II*) (C, H, M), *Desiccation protective protein* (*Le4*) (D, I, N), and *Pathogenesis-related protein 1a* (*PR1a*) (E, J, O) was measured in leaves of tomato plants without herbivores (Control), infected with *Meloidogyne incognita* (Mi) or *Spodoptera exigua* (Se) alone, or co-infected with both herbivores (MiSe). In co-infected plants, infestation with *S. exigua* was performed either at the nematode invasion (A–E), galling (F–J) or reproduction (K–O) stage. Samples were taken 24 h after *S. exigua* feeding. Data are the mean ±standard error (*n*=*5*). Different letters indicate significant differences between treatments, determined by Tukey’s HSD test for multiple comparisons after two-way ANOVA at *P*≤0.05.

**Fig. 4.**
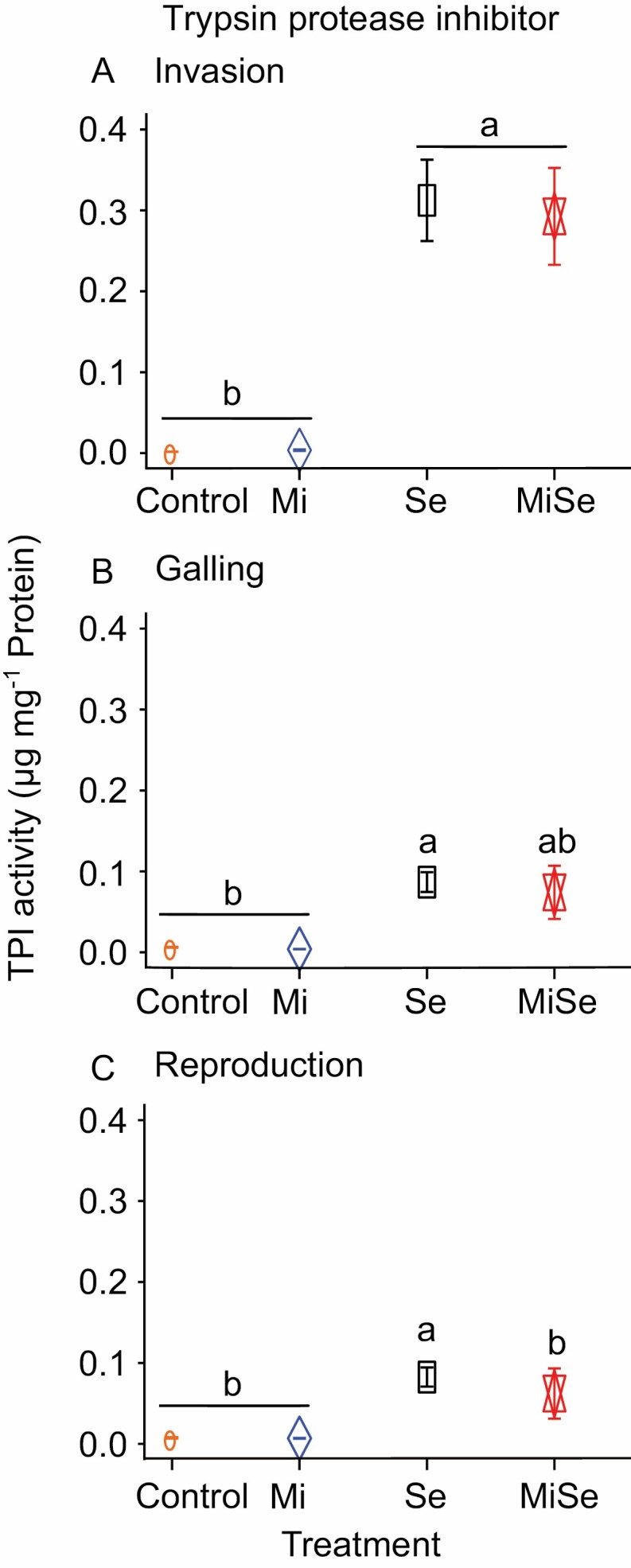
Trypsin protease inhibition activity in tomato leaves upon below- and aboveground herbivory. Trypsin protease inhibition activity was measured in leaves of tomato plants without herbivores (Control), infected with *Meloidogyne incognita* (Mi), or *Spodoptera exigua* (Se) alone, or co-infected with both herbivores (MiSe). In co-infected plants, infestation with *S. exigua* was performed either at the nematode invasion (A), galling (B), or reproduction (C) stage. Samples were taken 48 h after *S. exigua* feeding. Data are the mean ±standard error (*n*=5). Different letters indicate significant differences between treatments, determined by Tukey’s HSD test for multiple comparisons after two-way ANOVA at *P*≤0.05.

We next investigated whether *M. incognita* root infection influences the phytohormone-related responses triggered by *S. exigua* AG, at the different nematode root infection stages. We focused specifically on the JA-, SA-, and ABA-related pathways (Figs 2, 3). Our data indicate that when inoculated alone, *M. incognita* did not directly affect the endogenous concentration of 12-oxo-phytodienoic acid (OPDA), JA, JA-Ile, ABA, and SA in tomato leaves compared with controls, regardless of the infection cycle stage (Fig. 2, Mi versus Control treatment; Supplementary Table S3). Along the same lines, *M. incognita* infection did not directly affect the expression of the JA marker genes *Lipoxygenase D* (*LoxD*), *Prosystemin* (*PS*), and *Proteinase inhibitor II* (*PI II*); and neither the ABA marker gene *Desiccation protective protein* (*Le4*) nor the SA marker gene *Pathogenesis-related protein 1a* (*PR1a*) was affected (Fig. 3, Mi versus Control treatment; Supplementary Table S4). *Meloidogyne incognita* root infection did not directly affect the activity of trypsin protease inhibitor (TPI) (Fig. 4, Mi versus Control treatment; Supplementary Table S5) compared with control plants, regardless of the infection stage. These results suggest that *M. incognita* root infection, when inoculated alone, does not directly activate the JA-, SA- and ABA-related responses in tomato leaves.

*Spodoptera exigua* leaf-feeding resulted overall in a higher endogenous concentration of OPDA, JA, JA-Ile and ABA (Fig. 2A–D, F–I, K–N, Se versus Control treatment; Supplementary Table S3); and higher expression of the JA-related marker genes *LoxD*, *PS*, and *PI II*, and ABA-marker gene *Le4* (Fig. 3A–D, F–I, K–N, Se versus Control treatment; Supplementary Table S4). *Spodoptera exigua* leaf-feeding also led to increased endogenous SA levels in leaves (Fig. 2E, J, O, Se versus Control treatment; Supplementary Table S3), but it did not affect the expression of *PR1a* (Fig. 3E, J, O, Se versus Control treatment; Supplementary Table S4). Moreover, *Spodoptera exigua* leaf-feeding resulted in higher activity of TPI (Fig. 4, Se versus Control treatment; Supplementary Table S5).

In tomato plants co-infected with *M. incognita* and *S. exigua*, we found that at the nematode invasion stage (5 dpi), *M. incognita* root infection significantly reduced the endogenous levels of JA (*P*=0.009), ABA (*P*<0.001), and SA (*P*=0.006) in leaves, induced by *S. exigua* feeding (Fig. 2B, D, E, MiSe versus Se treatment; Supplementary Table S3). The increase in endogenous OPDA and JA-Ile levels triggered by *S. exigua* was only slightly attenuated on plants co-infected with both herbivores (Fig. 2A, C, MiSe versus Se treatment; Supplementary Table S3). Likewise, a significantly (*P*<0.001) lower expression of *PI II* was found in leaves of co-infected plants compared with that found in leaves of plants challenged with *S. exigua* alone (Fig. 3C, MiSe versus Se treatment; Supplementary Table S4). By contrast, *LoxD* and *PS* expression was higher in co-infected plants compared with the expression in plants challenged with *S. exigua* alone (Fig. 3A, B, MiSe versus Se treatment; Supplementary Table S4). There were no significant differences in the expression of *Le4* and *PR1a* in co-infected plants compared with plants infested with *S. exigua* alone (Fig. 3D,E, MiSe versus Se treatment; Supplementary Table S4). We did not find differences in the activity of TPI between co-infected plants and those challenged with *S. exigua* alone (Fig. 4A, MiSe versus Se treatment; Supplementary Table S5). These results indicate that at the invasion stage, the nematode *M. incognita* partially counteracts the *S. exigua*-triggered increase in the concentration of defense hormones. However, these hormonal responses were not fully connected with the expression level of their respective biosynthesis and responsive marker genes.

At the galling stage (15 dpi), we found a significant (*P*=0.02) increase in endogenous OPDA levels in co-infected plants compared with plants challenged with *S. exigua* alone (Fig. 2F, MiSe versus Se treatment; Supplementary Table S3). Correspondingly, a stronger expression of the JA biosynthesis marker gene *LoxD* was observed in co-infected plants, compared with plants challenged with *S. exigua* alone (Fig. 3F, MiSe versus Se treatment; Supplementary Table S4). There were no significant differences in the endogenous levels of JA, JA-Ile, and ABA between co-infected plants and plants challenged with *S. exigua* alone (Fig. 2G, H, I, MiSe versus Se treatment; Supplementary Table S3). Correspondingly, a similar expression level of *PS*, *PI II*, and *Le4* was found in co-infected plants and plants challenged with *S. exigua* alone (Fig. 3G, H, I, MiSe versus Se treatment; Supplementary Table S4). The endogenous concentrations of SA in co-infected plants were significantly (*P*=0.004) lower compared with the levels in plants infested with *S. exigua* alone (Fig. 2J, MiSe versus Se treatment; Supplementary Table S3). In contrast, *PR1a* expression level was similar in co-infected plants and in plants challenged with *S. exigua* alone (Fig. 3J, MiSe versus Se treatment; Supplementary Table S4). The activity of TPI in co-infected plants did not differ compared with plants challenged with *S. exigua* alone (Fig. 4B, MiSe versus Se treatment; Supplementary Table S5). In general, these observations indicate that at the galling stage, *M. incognita* enhances the *S. exigua*-triggered increase of OPDA concentration and *LOXD* expression, and partially counteracts the increase in SA concentrations triggered by *S. exigua*.

At the reproduction stage (30 dpi), a similar level of endogenous OPDA, JA, JA-Ile, ABA, and SA was found in co-infected plants and in plants challenged with *S. exigua* alone (Fig. 2K–O, MiSe versus Se treatment; Supplementary Table S3). In accordance, the expression of *LoxD*, *PS*, *PI II*, and *PR1a* in co-infected plants remained similar to that in plants challenged with *S. exigua* alone (Fig. 3K–M, O, MiSe versus Se treatment; Supplementary Table S4). A lower expression level of *Le4* was observed in co-infected plants compared with plants challenged with *S. exigua* alone (Fig. 3N, MiSe versus Se treatment; Supplementary Table S4). Notably, the activity of TPI in co-infected plants was significantly (*P*=0.006) reduced compared with plants challenged with *S. exigua* alone (Fig. 4C, MiSe versus Se treatment; Supplementary Table S5). Collectively, our data indicate that the *M. incognita* root infection can modulate systemically the JA-, ABA-, and SA-related responses elicited in leaves by *S. exigua* feeding. Our data further indicate that the effect of *M. incognita* on AG *S. exigua*-triggered responses varies depending on the nematode infection cycle stage.

### Root infection by *Meloidogyne incognita* systemically alters the carbon and nitrogen ratios in tomato leaves during the nematode galling stage

We tested whether root infection by *M. incognita* affects the concentrations of elemental C and N in tomato leaves. As shown in Table 2 and Supplementary Table S6, *M. incognita* root infection did not directly affect C and N concentrations in the leaves compared with controls, regardless of the infection cycle stage. Similarly, *S. exigua* herbivory did not affect C and N concentrations compared with control plants. The leaf C and N concentrations in co-infected plants remained similar to that observed in plants challenged with *S. exigua* alone.

**Table 2.** Concentrations of elemental carbon and nitrogen (as a percentage) and carbon/nitrogen ratio in tomato leaves upon below- and aboveground herbivory

Parameter	Treatment	Invasion	Galling	Reproduction
C	Control	40.80±0.30	42.65±0.75	43.13±0.69
	Mi	41.29±0.25	42.87±0.60	43.10±0.38
	Se	40.35±0.22	42.62±0.47	42.67±0.43
	MiSe	41.03±0.35	42.89±0.33	43.10±0.43
N	Control	4.74±0.25	3.62±0.17	2.62±0.30
	Mi	4.57±0.19	3.06±0.34	2.42±0.23
	Se	4.60±0.33	3.44±0.21	2.63±0.28
	MiSe	4.67±0.34	3.14±0.27	2.28±0.18
C/N ratio	Control	8.83±0.51	12.0±0.64	18.31±2.07
	Mi	9.17±0.41	**15.37±1.55**	19.57±2.39
	Se	9.21±0.76	12.79±0.82	17.74±1.95
	MiSe	9.21±0.74	14.51±1.28	20.04±1.90

Concentrations of elemental carbon (C) and nitrogen (N) (as a percentage), and the C/N ratio were determined in leaves of tomato plants without herbivores (control), infected with *Meloidogyne incognita* (Mi), or *Spodoptera exigua* (Se) alone, or co-infected with both herbivores (MiSe). In co-infected plants, infestation with *S. exigua* was performed at the nematode invasion, galling, or reproduction stage. Samples were taken 24 h after *S. exigua* feeding. Data are the mean ±standard error (*n*=3). Statistically significant means are indicated in bold.

In the case of the C/N ratio, we did not observe significant differences between plants infected with *M. incognita* at invasion (5 dpi) or reproduction (30 dpi) stage compared with control plants (Table 2; Supplementary Table S6). Notably, our data showed that *M. incognita* root infection at the galling stage (15 dpi) increased the C/N ratio in tomato leaves compared with control plants (Table 2; Supplementary Table S6). *Spodoptera exigua* herbivory did not affect C/N ratio compared with control plants (Table 2; Supplementary Table S6). The C/N ratio in co-infected plants remained similar to that observed in plants challenged with *S. exigua* alone throughout the nematode’s infection cycle (Table 2; Supplementary Table S6). These results show that root infection by *M. incognita* enhances the C/N ratio in leaves specifically when the *M. incognita* infection was at the galling stage (15 dpi).

### Root infection by *Meloidogyne incognita* at the galling stage alters the leaf metabolic profile triggered by *Spodoptera exigua* feeding

**Fig. 5.**
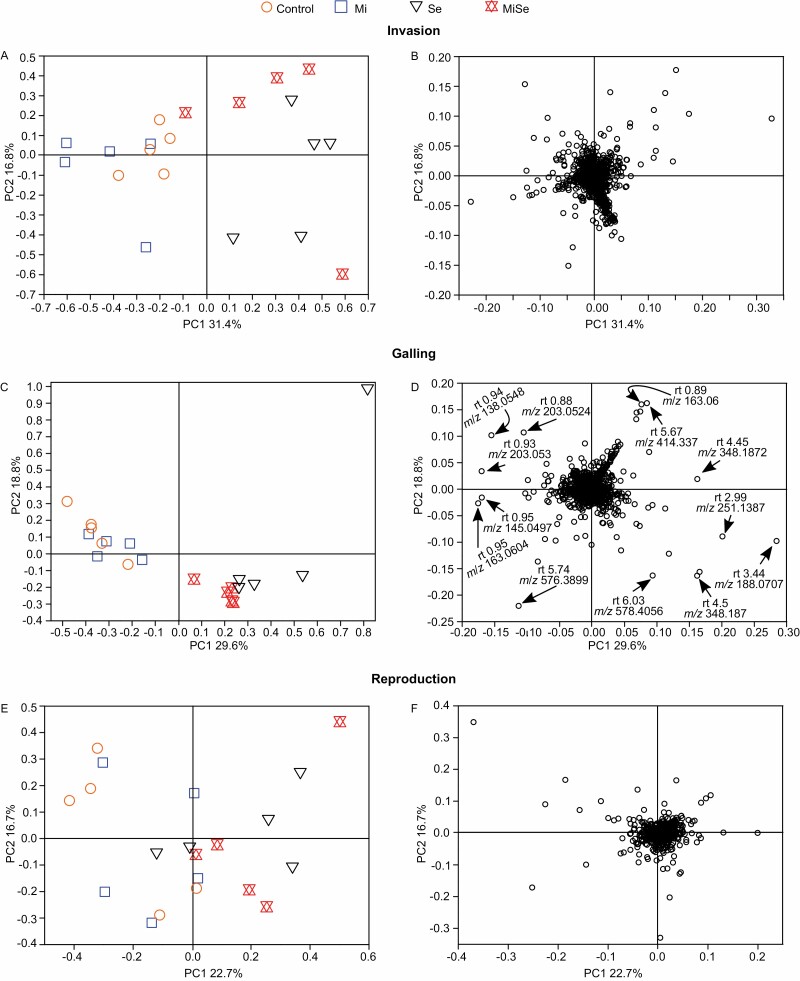
Principal component analysis: score and loading plots of leaf metabolic profiles in tomato plants upon below- and aboveground herbivory. Metabolic profiles analysed in leaves of tomato plants without herbivores (Control), infected with *Meloidogyne incognita* (Mi) or *Spodoptera exigua* (Se) alone, or co-infected with both herbivores (MiSe). In co-infected plants, infestation with *S. exigua* was performed at the nematode invasion (A, B), galling (C, D), or reproduction (E, F) stage. Samples were taken 24 h after *S. exigua* feeding. (A, C, E) Scores plots of principal components (PC) 1 and 2 showing the separation between the treatments. (B, D, F) Loading plots displaying the projection of each LC-MS feature. Arrows in (D) point to the most variable loadings selected for structural prediction.

We analysed the impact of *M. incognita* root infection at the invasion (5 dpi), galling (15 dpi), and reproduction (30 dpi) stages on the metabolic profile triggered in leaves by *S. exigua* feeding (Fig. 5). At the invasion stage (5 dpi), the first PC explained 31.4% of the total variance and revealed two clusters: control and *M. incognita*-infected plants in one group, and *S. exigua* and co-infected plants in the other group (Fig. 5A). At the galling stage (15 dpi), the first PC explained 29.6% of the total variance and revealed a separation of plants into two clusters: control and *M. incognita*-infected plants were all projected to the left while all plants treated with *S*. *exigua* were to the right of the score plot (Fig. 5C). In addition, we observed a separation between the co-infected plants from plants challenged with *S. exigua* alone. At the reproduction stage (30 dpi), the first two components explained 39.4% of the total variance, but we did not observe a clear separation between the groups (Fig. 5E). These results show that the impact of *S. exigua* feeding on the tomato leaf metabolome is stronger than the effect of *M. incognita* infection, at least during the invasion (5 dpi) and galling (15 dpi) stages. Our results further indicate that root infection by *M. incognita* partially alters the metabolic profiles triggered by *S. exigua* feeding, specifically during the galling stage (15 dpi).

### Root infection by *Meloidogyne incognita* alters the level of putative chemical defenses triggered by *Spodoptera exigua* feeding

**Fig. 6.**
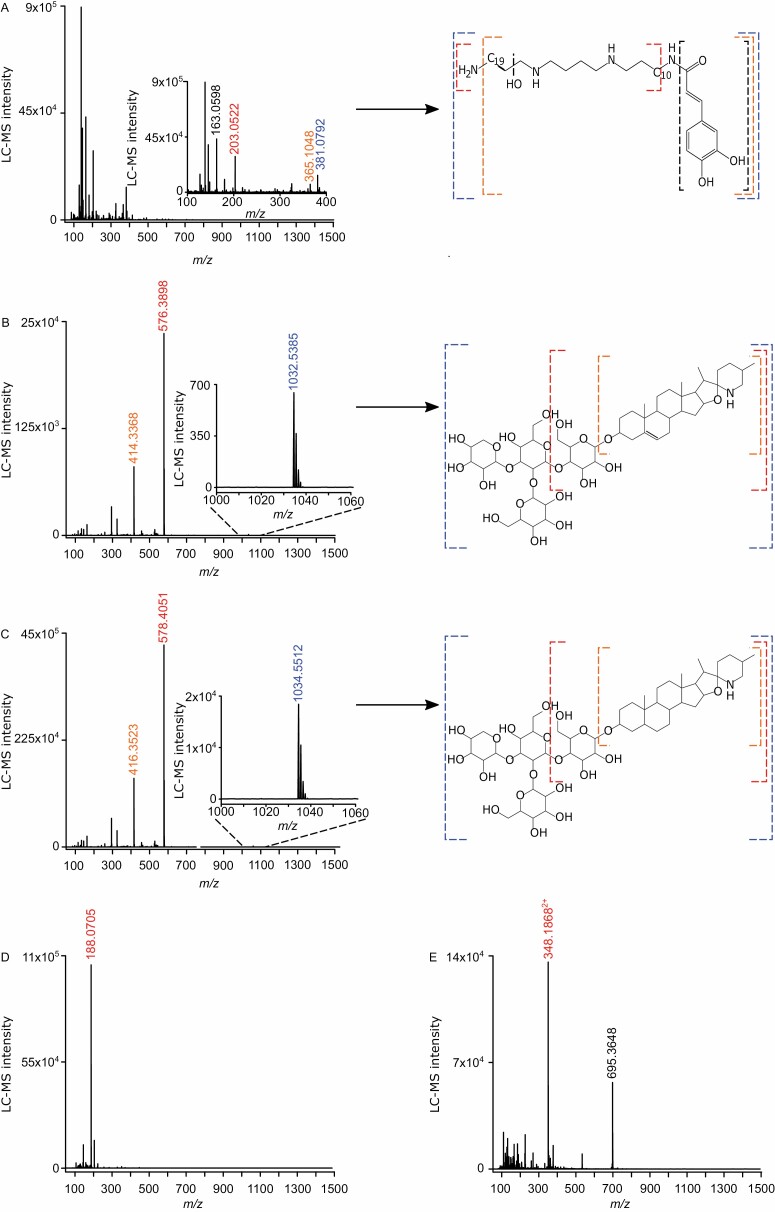
Mass spectra and structures of the predicted metabolites. Mass spectra and predicted structures of four highly variable metabolites selected at the nematode galling stage. Panels show the LC-MS intensities per metabolite detected by LC-MS in leaves of tomato plants without herbivores (Control), infected with *Meloidogyne incognita* (Mi), or *Spodoptera exigua* (Se) alone, or co-infected plants with both herbivores (MiSe). (A–C) Polyamine conjugated to a phenylpropanoid (A) and steroidal glycoalkaloids α*-*dehydrotomatine (B) and α-tomatine (C). (D, E) Two unknown metabolites that were among the most variable loadings. In (A–C) the numbers in blue represent the *m*/*z* of the predicted parent ion [M+H]^1+^, in red represent *m*/*z* of fragments that would have originated from the parent ion and are reported in the study, and in orange and black represent *m*/*z* of other fragments that likewise would have originated from the parent ion. In (D) the number in red represents the *m*/*z* value reported in the study. In (E) the number in black represents a *m*/*z* value, and the number in red its corresponding to *m*/2*z* (if *m*/*z* 695.3648 represents the molecular ion then *m*/*z* 348.1868 represents the species [M+2H]^2+^).

The effect of *M. incognita* root infection on *S. exigua* performance and the leaf metabolome was strongest at the nematode galling stage (15 dpi) (Figs 1, 5). For this reason, we analysed the metabolic profiles at the galling stage (15 dpi) in more detail. On the loadings plot of Fig. 5D, we selected the molecular features that were projected farthest from the center of the plot as they exhibit the highest variability and underlie the separation between the treatments found in Fig. 5C. Using the *m*/*z* value for each selected feature, we checked for signals in the chromatograms and picked out only the features with a conspicuous LC-MS peak and interpreted the mass spectra. We predicted structures of a polyamine conjugated to a phenylpropanoid with *m*/*z* 203.053 at 0.93 min retention time (rt) (Figs 5D, 6A), and two steroidal glycoalkaloids: α*-*dehydrotomatine with *m*/*z* 576.389 at 5.74 min rt, and α*-*tomatine with *m*/*z* 578.4056 at 6.03 min rt (Figs 5D, 6B, C). Two other selected features with *m*/*z* 188.0707 at 3.44 min rt and *m*/*z* 348.187 at 4.5 min rt had a conspicuous LC-MS peak, but we were unable to predict their structures (Figs 5D, 6D, E).

**Fig. 7.**
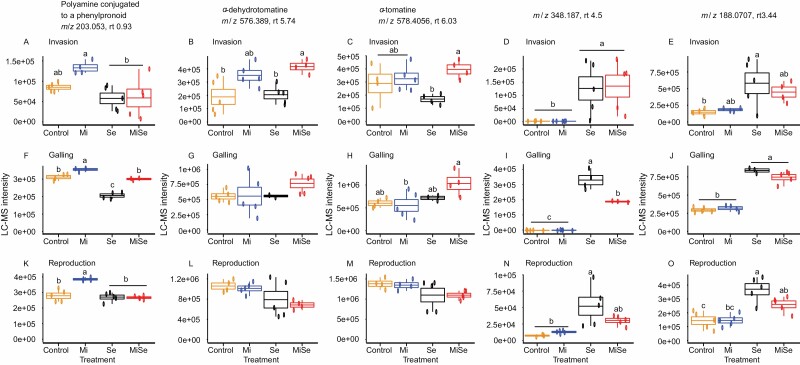
LC-MS intensities of the selected metabolites in tomato leaves upon below- and aboveground herbivory. LC-MS intensities, mass to charge ratio (*m*/*z*) and retention time (rt) in minutes of polyamine conjugated to a phenylpropanoid (A, F, K), α-dehydrotomatine (B, G, L), α-tomatine (C, H, M), and unknown metabolites (D, E, I, J, N, O) measured in leaves of tomato plants without herbivores (Control), infected with *Meloidogyne incognita* (Mi) or *Spodoptera exigua* (Se) alone, or co-infected with both herbivores (MiSe). In co-infected plants, infestation with *S. exigua* was performed at the nematode invasion, galling, or reproduction stage. Samples were taken 24 h after *S. exigua* feeding. Data are the mean ±standard error (*n*=5). Different letters indicate significant differences between treatments, determined by Tukey’s HSD test for multiple comparisons after two-way ANOVA at *P*≤0.05.

Next, we plotted the LC-MS intensities for the corresponding *m*/*z* values for both the predicted and the unknown metabolites selected at the galling stage (15 dpi) (Fig. 7F–J). To better understand the influence of the nematode’s life cycle on the *M. incognita*-triggered changes, we further plotted their LC-MS intensities using the datasets produced at the invasion (5 dpi) and reproduction stages (30 dpi) (Fig. 7A–E, K–O). We found that *M. incognita* root infection directly increased the leaf concentration of the polyamine conjugate throughout the entire infection cycle (Fig. 7A, F, K, Mi versus Control treatment; Supplementary Table S7). However, *M. incognita* root infection had no direct effect on the concentration of the steroidal glycoalkaloids α*-*dehydrotomatine and α*-*tomatine, nor on the two unknown metabolites (Fig. 7B–E, G–J, L–O, Mi versus Control treatment; Supplementary Table S7). Herbivory by *S. exigua*, however, triggered a decrease in the concentration of the polyamine conjugate in plants. This decrease coincided with the time point when the *M. incognita* infection was at the galling stage (15 dpi) (Fig. 7F, Se versus Control treatment; Supplementary Table S7). Herbivory by *S. exigua* alone did not affect the concentration of the steroidal glycoalkaloids compared with controls (Fig. 7B, C, G, H, L, M, Se versus Control treatment; Supplementary Table S7). However, *S. exigua* herbivory increased the concentration of the two unknown metabolites (Fig. 7D, E, I, J, N, O, Se versus Control treatment; Supplementary Table S7).

In tomato plants co-infected with *M. incognita* and *S. exigua*, we found that at the nematode invasion stage (5 dpi), the levels of the steroidal glycoalkaloids α*-*dehydrotomatine and α*-*tomatine were higher compared with the levels observed in plants challenged with *S. exigua* alone (Fig. 7B, C, MiSe versus Se treatment; Supplementary Table S7). However, in co-infected plants the levels of the polyamine conjugate and the unknown metabolites were similar to the levels observed in plants challenged with *S. exigua* alone (Fig. 7A, D–E, MiSe versus Se treatment; Supplementary Table S7). These results indicate that at the invasion stage, *M. incognita* counteracts the *S. exigua*-triggered repression of the steroidal glycoalkaloids.

At the galling stage (15 dpi) the concentration of the polyamine conjugate in co-infected plants increased compared with the concentration found in plants challenged with *S. exigua* alone (Fig. 7F, MiSe versus Se treatment; Supplementary Table S7). Co-infection did not affect the concentration of the steroidal glycoalkaloids or that of the unknown metabolite with *m*/*z* 188.0707 at 3.44 min rt compared with plants challenged with *S. exigua* alone (Fig. 7G, H, J, MiSe versus Se treatment; Supplementary Table S7). We observed that the concentration of the unknown metabolite with *m*/*z* 348.187 at 4.5 min rt was significantly (*P*<0.001) decreased in co-infected plants compared with plants challenged with *S. exigua* alone (Fig. 7I, MiSe versus Se treatment; Supplementary Table S7). These results show that at the galling stage, *M. incognita* counteracts the *S. exigua*-triggered repression of the polyamine conjugate, and partially impairs the enhancement of an unknown metabolite triggered by *S. exigua* feeding.

At the reproduction stage (30 dpi), we found that the levels of the selected metabolites in co-infected plants and in plants challenged with *S. exigua* alone were similar (Fig. 7K–O, MiSe versus Se treatment, Supplementary Table S7). All in all, our results indicate that the impact of *M. incognita* on the (putative) chemical defenses analysed differs according to the *M. incognita* life cycle stage.

## Discussion

Here, we demonstrate that the impact of the RKN *M. incognita* on the performance of the AG herbivore *S. exigua* is influenced by the nematode’s infection cycle. Our experimental design allowed us to identify that specifically at the galling stage, root infection by *M. incognita* enhanced the performance of the AG herbivore *S. exigua*. By contrast, *M. incognita* root infection did not affect the growth and the performance of *S. exigua* when the nematode was either at the invasion or reproduction stage (Fig. 1, Table 1). Previous studies have demonstrated the influence of RKNs on the performance of AG feeding herbivores (Carter-Wientjes *et al.*, 2004; Kaplan et al., 2008, 2009; Tiwari *et al.*, 2009; Arce *et al.*, 2017). Notably, these studies show a variety of interaction outcomes for the AG herbivores. For example, Kaplan *et al.* (2008) demonstrated that infection by *M. incognita* in tobacco roots increased the larval weight of the AG herbivores *Trichoplusia ni* and *S. exigua*, while it did not affect the performance of *M. sexta*. On the other hand, *M. incognita* root infection of soybean resulted in inconsistent effects on the performance of the AG herbivore *Pseudoplusia includens* (Carter-Wientjes *et al.*, 2004). These studies propose that the susceptibility of the host plant to the nematode infection and the identity of the herbivores are significant factors driving variation in the interaction outcomes for the AG herbivores (Wurst *et al.*, 2007; Sarmento *et al.*, 2011; Kyndt *et al.*, 2012; Wondafrash *et al.*, 2013; Biere and Goverse, 2016). Our findings point to the RKN infection cycle as a further key factor influencing the outcome of the interaction between RKN and AG herbivores when sharing a host plant. This is not surprising as the plant interaction with RKNs is highly dynamic, and root responses to RKNs profoundly differ between the initial and advanced stages of the infection cycle. For instance, by using the same biological system but focusing on roots, we recently found that *M. incognita* infection triggers a defensive response in tomato roots specifically at the reproduction stage. This response involves the JA-, SA- and ABA-pathways and glycoalkaloids (Mbaluto *et al.*, 2020). Such differences in root responses over the infection cycle of the nematode may lead to different systemic responses, and thereby have different effects on insect herbivores feeding on AG plant tissues. The enhanced *S. exigua* performance on *M. incognita* root-infected plants at the galling stage was not accompanied by a higher leaf consumption (Supplementary Fig. S2). This indicates that the facilitation by *M. incognita* at the galling stage may have been mediated by an increase in leaf nutritional quality or by a suppression of the plant’s ability to mount an effective defense against *S. exigua*.

We next aimed to shed some light on the mechanisms that might underlie *M. incognita*’s facilitation of *S. exigua* performance at the galling stage. Although *M. incognita* when inoculated alone did not directly alter JA-, SA-, or ABA-related pathways AG, it did affect the phytohormone-related responses triggered in leaves by *S. exigua* feeding (Figs 2, 3). Interestingly, the modulation of the *S. exigua*-induced phytohormone pathways by *M. incognita* varied over the nematode’s infection cycle, being most evident when *M. incognita* was at the invasion and galling stages. For instance, regarding the JA-related pathway, *M. incognita* root infection at the invasion stage impaired the accumulation of JA and the transcriptional activation of *PI II* triggered by *S. exigua* feeding, suggesting the ability of *M. incognita* to repress the JA-related response triggered AG by *S. exigua* feeding (Figs 2, 3). By contrast, at the galling stage, *M. incognita* enhanced the accumulation of OPDA and the expression of the JA-related biosynthesis gene *LOXD* elicited by *S. exigua* (Figs 2, 3), pointing to a priming effect by *M. incognita* infection on the JA biosynthesis pathway (Martínez-Medina *et al.*, 2016). Moreover, *M. incognita* root infection at the reproduction stage enhanced the expression of *PI II* triggered by *S. exigua*, while it reduced the activity of TPI elicited by *S. exigua* (Figs 3, 4). The fact that the analysis of TPI activity was performed later (48 h after *S. exigua* herbivory) than the phytohormonal and transcriptomic analyses (24 h after *S. exigua* herbivory) renders it difficult to directly relate these datasets. Strikingly, the systemic modulation of the leaf JA pathway by *M. incognita* did not correlate with the performance of *S. exigua*, even though the JA pathway is one of the central pathways governing plant defenses against AG chewing herbivores (Erb *et al.*, 2012; Wasternack and Strnad, 2016). Indeed, we found a facilitation effect on *S. exigua* by *M. incognita* infection at the galling stage, which concurred with enhanced accumulation of the JA precursor OPDA and the JA biosynthesis marker gene *LoxD*. OPDA contributes to plant resistance to herbivory, independently of the JA/JA-Ile biosynthesis and signaling (Bosch et al., 2014*a*, *b*). However, consistent with our observations, it was demonstrated that OPDA-mediated induction of resistance is not sufficient for conferring plant resistance to *S. exigua* herbivory (Bosch *et al.*, 2014*b*). On the other hand, the impairment of *S. exigua*-triggered JA accumulation and *PI II* expression elicited by *M. incognita* at the invasion stage was not accompanied by any effect on *S. exigua* performance. This may suggest that JA-triggered *PI II* does not have a major role in the performance of *S. exigua*. Along the same lines, Jongsma *et al.* (1995) found that *S. exigua* growth was unaffected by high expression levels of *PI II* in tobacco leaves. Altogether, these findings suggest the existence of additional mechanisms underlying the impact of root infection by *M. incognita* on the performance of *S. exigua*.

Besides the JA pathway, the SA and ABA pathways are important players in the orchestration of plant defenses against herbivorous insects (Diezel *et al.*, 2009; Erb *et al.*, 2009). Indeed, the negative crosstalk between the SA and JA pathways is proposed to regulate plant resistance to *S. exigua* (Cipollini *et al.*, 2004; Diezel *et al.*, 2009). We found that *S. exigua* feeding triggered foliar SA accumulation (Fig. 2). Interestingly, such increase was less pronounced in plants that were also infected with *M. incognita* at the invasion and galling stages. It has been proposed that some insect herbivores such as *S. exigua* can enhance their fitness by activating the SA pathway to weaken JA-mediated defenses (Diezel *et al.*, 2009). However, our study did not evidence negative crosstalk between the JA and SA pathways. Moreover, besides the reduced SA levels mediated by co-infection at the invasion and galling stages, *S. exigua* performed better when feeding on *M. incognita*-infected plants at the galling stage. This suggests that further hormone pathways could be involved in the *M. incognita* facilitation of *S. exigua* performance. The ABA pathway is involved in the rewiring of JA-dependent defenses during herbivory (Van Poecke, 2007). Indeed, ABA-deficient mutants are more susceptible to herbivory (Thaler and Bostock, 2004; Vos *et al.*, 2013). We found that *M. incognita* at the invasion stage decreased the *S. exigua*-triggered increase in endogenous ABA levels (Fig. 2). However, this effect was not correlated with changes in the performance of *S. exigua*. Overall, these findings suggest the existence of additional mechanisms underlying the impact of root infection by *M. incognita* on the performance of *S. exigua*.

Metabolomics approaches provide an opportunity to assess local and systemic herbivore-induced changes in plant metabolic patterns without any prior assumption (Viant, 2008; Peters *et al.*, 2018). We applied untargeted metabolomics to assess whether *M. incognita* root infection altered the leaf metabolome elicited by *S. exigua* herbivory and whether this effect was modulated by the nematode’s infection cycle. We found a stronger impact of *S. exigua* herbivory on the tomato leaf metabolome compared with the impact of *M. incognita* root infection (Fig. 5). Moreover, the leaf metabolic profiles triggered by *S. exigua* herbivory were markedly different from those triggered by *M. incognita* root infection, especially at the invasion and galling stages. Although the identity of the metabolites altered in both interactions remains unknown, such differences may underlie the different feeding styles and life strategies of both herbivores (Wondafrash *et al.*, 2013).

While the direct impact of root infection by *M. incognita* on leaf metabolic profiles was moderate, *M. incognita* altered at least partially the metabolic profiles triggered by *S. exigua* herbivory, at the nematode invasion and galling stages (Fig. 5). Our results demonstrated that *M. incognita* at the galling stage enhanced *S. exigua* performance. Therefore, using the loading plot of the galling stage we selected and predicted the metabolites that might underlie the observed phenotype across the *M. incognita* infection stages. Among the LC-MS features with the highest variability in the PCA, we predicted a polyamine conjugated to a phenylpropanoid (Fig. 6). Although further analysis would be required, we suggest that, according to its mass spectrum, it may be a derivative of spermine. Polyamine conjugates have been shown to have a prominent role in plant defense against herbivores. Accumulation of putrescine/spermidine polyamine conjugates was strongly induced in tobacco plants by herbivory, and this is coordinated by the transcription factor MYB8 (Kaur *et al.*, 2010; Onkokesung *et al.*, 2012). Moreover, *M. sexta* and *S. littoralis* feeding on systemically pre-induced leaves performed significantly better on ir-MYB8 plants lacking phenylpropanoid–polyamine conjugates compared with wild-type plants expressing high levels of phenylpropanoid–polyamine conjugates (Kaur *et al.*, 2010). Remarkably, *S. exigua* feeding led to a decrease in the concentration of the predicted polyamine conjugate (Fig. 7). This decrease might be related to the ability of *S. exigua* to down-regulate plant immune responses (Bandoly *et al.*, 2015). In contrast, *M. incognita* root infection stimulated the accumulation of this polyamine conjugate in leaves, throughout the entire infection cycle. Plant-parasitic nematodes can manipulate the biosynthesis of polyamines to promote infection (Hewezi *et al.*, 2010). Remarkably, at the galling stage, *M. incognita* root infection counteracted the decrease in the concentration of the detected polyamine conjugate triggered by *S. exigua* feeding. Taking into consideration that we found a facilitation effect of *M. incognita* on the performance of *S. exigua*, we hypothesize that the predicted polyamine conjugate does not play a major role in plant defenses against *S. exigua*. The polyamine biosynthetic pathway is highly interconnected and plastic, leading to the biosynthesis of a broad spectrum of polyamine conjugates depending on the specific stress (Kaur *et al.*, 2010; Onkokesung *et al.*, 2012). It was further suggested that a mixture of various polyamine conjugates may be required to exert the maximal efficiency of polyamine conjugates against herbivores (Onkokesung *et al.*, 2012). Whereas further studies are required to shed more information on the role of polyamines and their conjugates in AG–BG interactions, we hypothesize that this specific polyamine conjugate does not play a major role in the facilitation effect triggered by *M. incognita* on *S. exigua* performance.

Besides the polyamine conjugate, we also found the steroidal glycoalkaloids α-dehydrotomatine and α-tomatine to be affected by co-infection (Fig. 6). Individually, neither *M. incognita* root infection nor *S. exigua* feeding affected the accumulation of these steroidal glycoalkaloids in tomato leaves (Fig. 7). However, in leaves of co-infected plants where *M. incognita* was at the invasion stage, the accumulation of these steroidal glycoalkaloids was higher compared with leaves of plants challenged with *S. exigua* alone. Moreover, we found a similar trend when *M. incognita* root infection was at the galling stage.

Steroidal glycoalkaloids in *Solanum* species function as first-line defense metabolites against pathogens and herbivores (Güntner *et al.*, 1997; Friedman, 2002; Ökmen *et al.*, 2013; Chowański *et al.*, 2016; Carere *et al.*, 2016; Dahlin *et al.*, 2017; Garcia *et al.*, 2018). Despite the observed increase in steroidal glycoalkaloid concentration in the co-infected plants, we did not detect negative effects on the performance of *S. exigua*. In fact, we found that *S. exigua* larvae performed better in the co-infected plants at the galling stage of *M. incognita*. Secondary metabolites can vary in their effects on insect herbivores. For example, in potato the accumulation of the steroidal glycoalkaloids α*-*solanine and α*-*chaconine reduces *S. exigua* growth (Kumar *et al.*, 2016), while in black nightshade it does not affect the phytophagous lady beetle *Henosepilachna vigintioctomaculata* (Hori *et al.*, 2011). Interestingly, a previous study demonstrated that α-tomatine had little or no effect on food consumption, assimilation, or dietary utilization by *S. exigua* larvae and other herbivores (Bloem *et al.*, 1989). These studies demonstrate that steroidal glycoalkaloids can vary in their effects on insect herbivores. In our case, the results indicate that the stronger accumulation of the steroidal glycoalkaloids in co-infected plants did not affect the performance of *S. exigua*.

Among the most variable molecular features were also two metabolites with *m*/*z* 188.0707 at 3.44 min rt and *m*/*z* 348.187 at 4.5 min rt (Fig. 6). We found that *S. exigua* feeding led to an enhanced accumulation of these metabolites (Fig. 7). Although we were unable to predict the structures of these metabolites, we hypothesize that they might act as induced anti-herbivory defense compounds. It is remarkable that when *M. incognita* was at the galling stage, it partially counteracted the *S. exigua*-triggered accumulation of the metabolite with *m*/*z* 348.187 at 4.5 min rt. Noticeably, this effect exerted by *M. incongita* infection was specific to the galling stage. Therefore, we hypothesize that this effect might underlie, at least partially, the facilitation effect observed specifically at the nematode’s galling stage.

Besides the changes in plant defense traits, the performance and population dynamics of AG insect herbivores also depend on the nutritive quality of the host plant (Awmack and Leather, 2002). It has been established that after herbivory, plants allocate C and N to specific tissues to be utilized for compensatory growth or defense of valuable plant parts (Creelman and Mullet, 1997; Wang *et al.*, 2016; Kafle *et al.*, 2017). Our results showed that *M. incognita* did not affect elemental C and N content in leaves, but it did increase the C/N ratio, specifically at the nematode’s galling stage (Table 2). Moreover, at the nematode’s galling stage, we observed a higher (although not statistically significant) C/N ratio in co-infected plants compared with plants challenged with *S. exigua* alone. It is established that higher C/N ratios in plant tissues generally reduce host plant quality for herbivores (Bryant *et al.*, 1983; Luo *et al.*, 2006; Dáder *et al.*, 2016). However, we found an enhanced performance of *S. exigua* when feeding on plants infected by the nematode at the galling stage. We therefore speculate that this potential reduction in host plant quality mediated by *M. incognita* did not contribute to the facilitation effect observed.

In conclusion, our findings demonstrate that the impact of root infection by the RKN *M. incognita* on systemic defense responses and the performance of the AG herbivore *S. exigua* significantly varied over the nematode’s root infection cycle. Our results further suggest that the specific leaf responses triggered systemically by *M. incognita* at each of the different life cycle stages underlie the differential impact of *M. incognita* throughout its life cycle on plant resistance to *S. exigua*. We propose that it is crucial to consider the root infection cycle of the RKN *M. incognita* in future studies dealing with AG–BG plant-mediated interactions.

## Supplementary data

The following supplementary data are available at *JXB* online.

Fig. S1. Number of *Meloidogyne incognita* galls in tomato roots.

Fig. S2. Amount of leaf material consumed by *Spodoptera exigua*.

Table S1. List of primer sequences used for qRT-PCR reactions.

Table S2. Student’s *t-*test results for the performance of *Spodoptera exigua* feeding on *Meloidogyne incognita*-infected plants.

Table S3. ANOVA results for the concentrations of phytohormones in tomato leaves upon below- and aboveground herbivory.

Table S4. ANOVA results for the expression of marker genes in defense signaling pathways in tomato leaves upon below- and aboveground herbivory.

Table S5. ANOVA results for the trypsin protease inhibitor activity in tomato leaves upon below- and aboveground herbivory.

Table S6. ANOVA results for the concentrations of elemental carbon and nitrogen (as a percentage) and carbon/nitrogen ratio in tomato leaves upon below- and aboveground herbivory.

Table S7. ANOVA results for the LC-MS intensities of the selected metabolites in tomato leaves upon below- and aboveground herbivory.

erab370_suppl_Supplementary_Figures_S1-S2_Tables_S1-S7

## Data Availability

The datasets underlying this study are openly available at the iDiv data repository. Dataset on the performance of *Spodoptera exigua*: https://doi.org/10.25829/idiv.1839-15-1031; dataset on plant defense responses induced during herbivory: https://doi.org/10.25829/idiv.1833-20-1029; Mbaluto *et al.* (2021).
