# Supplementary material for: Root infection by the nematode Meloidogyne incognita modulates leaf antiherbivore defenses and plant resistance to Spodoptera exigua
Source: J Exp Bot. 2021 Sep 21;72(22):7909–26. doi: 10.1093/jxb/erab370 (PMC8664589; doi:10.1093/jxb/erab370)
Supplement: erab370_suppl_Supplementary_Figures_S1-S2_Tables_S1-S7 [file erab370_suppl_supplementary_figures_s1-s2_tables_s1-s7.pdf]

**Root infection by the nematode *Meloidogyne incognita* modulates leaf antiherbivore defenses and plant resistance to *Spodoptera exigua***

Crispus M. Mbaluto, Fredd Vergara, Nicole M. van Dam, Ainhoa Martínez-Medina,

**Supplementary Data**

**Table S1** List of primer sequences used for qRT PCR reactions.

**Table S2** Student's *t*-test results for the performance of *Spodoptera exigua* feeding on *Meloidogyne incognita* infected plants.

**Table S3** ANOVA results for the concentrations of phytohormones in tomato leaves upon below- and aboveground herbivory.

**Table S4** ANOVA results for the expression of marker genes in defense signaling pathways in tomato leaves upon below- and aboveground herbivory.

**Table S5** ANOVA results for the trypsin protease inhibitor activity in tomato leaves upon below- and aboveground herbivory.

**Table S6** ANOVA results for the concentrations of elemental carbon and nitrogen (in percentages), and carbon/nitrogen ratio in tomato leaves upon below- and aboveground herbivory.

**Table S7** ANOVA results for the LC-MS intensities of the selected metabolites in tomato leaves upon below- and aboveground herbivory.

**Fig. S1** Number of *Meloidogyne incognita* galls/knots in tomato roots.

**Fig. S2** Amount of leaf material consumed by *Spodoptera exigua*.

**Table S1 List of primer sequences used for qRT PCR reactions.**

| Target gene | Related pathway | Primer sequences (5'→3') |
|---|---|---|
| *Lipoxygenase D* (*LoxD*)[a] | JA inducible | Fw: GGCTTTATTTCACACAGAGATA<br>Rev: ATGTGCTGCCAATATAAATGGTTCC |
| *Prosystemin* (*PS*)[b] | | Fw: AATTTGTCTCCCGTTAGA<br>Rev: AGCCAAAAGAAAGGAAGCAAT |
| *Proteinase inhibitor II* (*PI II*)[b] | | Fw: GAAAATCGTTAATTTATCCCAC<br>Rev: ACATACAAACTTTCCATCTTTA |
| Desiccation protective protein (*Le4*)[b] | ABA inducible | Fw: GAAGTTGCCACCATGTAAGG<br>Rev: ACTCAAGGCATGGGTACTGG |
| *Pathogenesis-related protein 1a* (*PR1a*)[b] | SA inducible | Fw: GTGGGATCGGATTGATATCCT<br>Rev: CCTAAGCCACGATACCATGAA |
| *Elongation factor 1α* (*SlEF*)[b] | Housekeeping | Fw: GATTGGTGGTATTGGAACTGTC<br>Rev: AGCTTCGTGGTGCATCTC |

[§];Fw: forward, Rev: reverse.

[a]**Uppalapati SR, Ayoubi P, Weng H, Palmer DA, Mitchell RE, Jones W, Bender CL**. 2005. The phytotoxin coronatine and methyl jasmonate impact multiple phytohormone pathways in tomato. The Plant Journal **42**, 201–217.

[b]**Martínez-Medina A, Fernández I, Sánchez-Guzmán MJ, Jung SC, Pascual JA, Pozo MJ**. 2013. Deciphering the hormonal signalling network behind the systemic resistance induced by *Trichoderma harzianum* in tomato. Frontiers in Plant Science **4**, 1–12.

**Table S2 Student's *t*-test results on the performance of *Spodoptera exigua* feeding on *Meloidogyne incognita* infected plants*.*** *Spodoptera exigua* performance was assessed through larval weight gain, pupal weight, and pupation time. These performance indicators were measured from larvae feeding on leaves of control plants (without root infection) and leaves of plants infected in roots with *Meloidogyne incognita* (Mi) either at the invasion, galling, and reproduction stages. The data were analyzed for each performance indicator using student *t*-test*,* and statistically significant effects inferred at $P \leq 0.05$ and are indicated in bold.

| Performance indicator | Invasion | | | Galling | | | Reproduction | | |
|---|---|---|---|---|---|---|---|---|---|
| | Df | T | P | Df | T | P | Df | T | P |
| Larval weight (mg) | 12 | -0.027 | 0.979 | 10 | 0.272 | 0.791 | 10 | -0.100 | 0.922 |
| Pupal weight (mg) | 16 | 0.327 | 0.748 | 28 | 1.986 | **0.057** | 15 | -0.079 | 0.938 |
| Pupation time (d) | 15 | 0.127 | 0.901 | 21 | -3.404 | **0.003** | 14 | 0.966 | 0.350 |

[§]; Df: degree of freedom, T: T-statistics value, P: probability value, mg: milligram, d: day(s).

**Table S3 ANOVA results for the concentrations of phytohormones in tomato leaves upon below- and aboveground herbivory**. Concentrations of 12-oxo-phytodienoic acid (OPDA), jasmonic acid (JA), and jasmonyl-L-isoleucine (JA-Ile), abscisic acid (ABA) and salicylic acid (SA) were measured in leaves of tomato plants without herbivores (Control), infected with *Meloidogyne incognita* (Mi) or *Spodoptera exigua* (Se) alone, or double infected with both herbivores (MiSe). In double infected plants, infestation with *S. exigua* was performed either at the nematode's invasion, galling, or reproduction stages. Samples were taken 24 hours after *S. exigua* feeding. Data were analyzed using a Two-way ANOVA linear model consisting of *M. incognita* (Mi), *S. exigua* (Se), and their interaction (Mi*Se) as model explanatory factors. The differences between the treatments were detected by Tukey's HSD test for multiple comparisons at $P \leq 0.05$. Statistically significant effects are indicated in bold.

| Hormone | Source of variation | Invasion | | | Galling | | | Reproduction | | |
|---|---|---|---|---|---|---|---|---|---|---|
| | | $Df_{(n,d)}$ | F | P | $Df_{(n,d)}$ | F | P | $Df_{(n,d)}$ | F | P |
| OPDA | Mi | 1,29 | 1.9919 | 0.169 | 1,14 | 16.6384 | **0.001** | 1,14 | 2.2006 | 0.160 |
| | Se | 1,29 | 55.2256 | **<0.001** | 1,14 | 36.4689 | **<0.001** | 1,14 | 0.8498 | 0.372 |
| | Mi*Se | 1,29 | 2.1977 | 0.149 | 1,14 | 6.9356 | **0.020** | 1,14 | 0.2507 | 0.624 |
| JA | Mi | 1,30 | 6.7808 | **0.014** | 1,14 | 0.1724 | 0.684 | 1,15 | 3.3054 | 0.089 |
| | Se | 1,30 | 54.7066 | **<0.001** | 1,14 | 102.9905 | **<0.001** | 1,15 | 2.1413 | 0.164 |
| | Mi*Se | 1,30 | 7.7520 | **0.009** | 1,14 | 0.1278 | 0.726 | 1,15 | 3.1070 | 0.098 |
| JA-Ile | Mi | 1,28 | 0.7736 | 0.387 | 1,13 | 0.5259 | 0.481 | 1,15 | 3.2780 | 0.090 |
| | Se | 1,28 | 48.5152 | **<0.001** | 1,13 | 112.9965 | **<0.001** | 1,15 | 2.2171 | 0.157 |
| | Mi*Se | 1,28 | 0.6792 | 0.417 | 1,13 | 0.4483 | 0.515 | 1,15 | 2.2519 | 0.154 |
| ABA | Mi | 1,27 | 10.436 | **0.003** | 1,13 | 6.0747 | **0.028** | 1,16 | 0.0456 | 0.834 |
| | Se | 1,27 | 43.994 | **<0.000** | 1,13 | 80.4095 | **<0.000** | 1,16 | 2.3664 | 0.144 |
| | Mi*Se | 1,27 | 19.772 | **0.000** | 1,13 | 3.6685 | 0.078 | 1,16 | 0.3385 | 0.569 |
| SA | Mi | 1,26 | 11.3872 | **0.023** | 1,12 | 0.7664 | 0.399 | 1,15 | 1.6700 | 0.216 |
| | Se | 1,26 | 22.1075 | **<0.000** | 1,12 | 12.7078 | **0.004** | 1,15 | 3.4739 | 0.082 |
| | Mi*Se | 1,26 | 9.0112 | **0.006** | 1,12 | 0.1449 | 0.710 | 1,15 | 0.4075 | 0.533 |

[§]; Df: degree of freedom, (n,d): numerator and denominator of Df, F: F-statistics value, P: probability value.

**Table S4 ANOVA results for the expression of marker genes in defense signaling pathways in tomato leaves upon below- and aboveground herbivory.** Transcript expression levels of *Lipoxygenase D* (*LoxD*)*, Prosystemin* (*PS*)*, Proteinase inhibitor II* (*PI II*) *Desiccation protective protein* (*Le4*), and *Pathogenenis-related protein 1a* (*PR1a*) were measured in leaves of tomato plants without herbivores (Control), infected with *Meloidogyne incognita* (Mi) or *Spodoptera exigua* (Se) alone, or double infected with both herbivores (MiSe). In double infected plants, infestation with *S. exigua* was performed either at the nematode's invasion, galling, or reproduction stages. Samples were taken 24 hours after *S. exigua* feeding. Data were analyzed using a Two-way ANOVA linear model consisting of *M. incognita* (Mi), *S. exigua* (Se), and their interaction (Mi*Se) as model explanatory factors. The differences between the treatments were detected by Tukey's HSD test for multiple comparisons at $P \leq 0.05$. Statistically significant effects are indicated in bold.

| Marker genes | Source of variation | Invasion | | | Galling | | | Reproduction | | |
|---|---|---|---|---|---|---|---|---|---|---|
| | | $Df_{(n,d)}$ | F | P | $Df_{(n,d)}$ | F | P | $Df_{(n,d)}$ | F | P |
| | Mi | 1,31 | 35.359 | **<0.001** | 1,27 | 9.1802 | **0.005** | 1,23 | 0.5436 | 0.468 |
| *LoxD* | Se | 1,31 | 35.592 | **<0.001** | 1,27 | 334.0329 | **<0.001** | 1,23 | 29.1066 | **<0.001** |
| | Mi*Se | 1,31 | 33.397 | **<0.001** | 1,27 | 8.4198 | **0.007** | 1,23 | 0.6225 | 0.438 |
| | Mi | 1,28 | 10.8804 | **0.003** | 1,26 | 0.1394 | 0.712 | 1,26 | 0.3023 | 0.587 |
| *PS* | Se | 1,28 | 364.7633 | **<0.001** | 1,26 | 44.8585 | **<0.001** | 1,26 | 42.1531 | **<0.001** |
| | Mi*Se | 1,28 | 1.9591 | 0.173 | 1,26 | 0.0212 | 0.885 | 1,26 | 2.2918 | 0.142 |
| | Mi | 1,29 | 13.902 | **0.001** | 1,24 | 0.9507 | 0.339 | 1,25 | 3.9052 | 0.059 |
| *PI II* | Se | 1,29 | 38.258 | **<0.001** | 1,24 | 8.3204 | **0.008** | 1,25 | 14.7587 | **0.001** |
| | Mi*Se | 1,29 | 13.153 | **0.001** | 1,24 | 0.8216 | 0.374 | 1,25 | 3.6538 | 0.067 |
| | Mi | 1,30 | 3.7989 | 0.0607 | 1,19 | 0.0030 | 0.9568 | 1,18 | 2.6268 | 0.1225 |
| *Le4* | Se | 1,30 | 15.0082 | **0.0005** | 1,19 | 38.9801 | **5.38e-06** | 1,18 | 0.3398 | 0.5672 |
| | Mi*Se | 1,30 | 1.8348 | 0.1857 | 1,19 | 0.0258 | 0.8741 | 1,18 | 7.3520 | **0.0143** |
| | Mi | 1,29 | 3.2087 | 0.0837 | 1,19 | 0.9549 | 0.3408 | 1,18 | 0.0546 | 0.8179 |
| *PR1a* | Se | 1,29 | 11.7607 | **0.0018** | 1,19 | 0.1711 | 0.6838 | 1,18 | 0.4352 | 0.5178 |
| | Mi*Se | 1,29 | 11.5595 | **0.0020** | 1,19 | 6.4400 | **0.0201** | 1,18 | 0.3389 | 0.5677 |

§; Df: degree of freedom, (n,d): numerator and denominator of Df, F: F-statistics value, P: probability value.

**Table S5 ANOVA results for the trypsin protease inhibitor activity in tomato leaves upon below- and aboveground herbivory.** The activity of trypsin proteases was determined in leaves of tomato plants without herbivores (Control), infected with *Meloidogyne incognita* (Mi), or *Spodoptera exigua* (Se) alone, or double infected with both herbivores (MiSe). In double infected plants, infestation with *S. exigua* was performed either at the nematode's invasion, galling, or reproduction stages. Samples were taken 48 hours after *S. exigua* feeding. Data were analyzed using a Two-way ANOVA linear model consisting of *M. incognita* (Mi), *S. exigua* (Se), and their interaction (Mi*Se) as model explanatory factors. The differences between the treatments were detected using Tukey's HSD test for multiple comparisons at $P \leq 0.05$. Statistically significant effects are indicated in bold.

| Nematode's infection stage | Source of variation | Statistics | | |
|---|---|---|---|---|
| | | $Df_{(n,d)}$ | F | P |
| Invasion | Mi | 1,16 | 0.0528 | 0.821 |
| | Se | 1,16 | 58.7901 | **<0.001** |
| | Mi*Se | 1,16 | 0.0763 | 0.786 |
| Galling | Mi | 1,16 | 0.1820 | 0.675 |
| | Se | 1,16 | 18.4215 | **0.001** |
| | Mi*Se | 1,16 | 0.0881 | 0.770 |
| Reproduction | Mi | 1,15 | 9.5735 | **0.007** |
| | Se | 1,15 | 44.5982 | **<0.001** |
| | Mi*Se | 1,15 | 10.2532 | **0.006** |

§; Df: degree of freedom, (n,d): numerator and denominator of Df, F: F-statistics value, P: probability value.

**Table S6 ANOVA results for the concentrations of elemental carbon and nitrogen (in percentages), and carbon/nitrogen ratio in tomato leaves upon below- and aboveground herbivory.** The concentrations of carbon (C), nitrogen (N), and carbon/nitrogen (C/N) ratio were determined in leaves of tomato plants without herbivores (Control), infected with *Meloidogyne incognita* (Mi), or *Spodoptera exigua* (Se) alone, or double infected with both herbivores (MiSe). In double infected plants, infestation with *S. exigua* was performed either at the nematode's invasion, galling, or reproduction stages. Samples were taken 24 hours after *S. exigua* feeding. Data were analyzed using a Two-way ANOVA linear model consisting of *M. incognita* (Mi), *S. exigua* (Se), and their interaction (Mi*Se) as model explanatory factors. The differences between the treatments were detected by Tukey's HSD test for multiple comparisons at $P \leq 0.05$. Statistically significant effects are indicated in bold.

| Parameter | Source of variation | Invasion | | | Galling | | | Reproduction | | |
|---|---|---|---|---|---|---|---|---|---|---|
| | | $Df_{(n,d)}$ | F | P | $Df_{(n,d)}$ | F | P | $Df_{(n,d)}$ | F | P |
| C | Mi | 1,29 | 1.3330 | 0.258 | 1,32 | 0.1946 | 0.662 | 1,32 | 0.1474 | 0.704 |
| | Se | 1,29 | 1.2932 | 0.265 | 1,32 | 0.0000 | 1.000 | 1,32 | 0.2192 | 0.649 |
| | Mi*Se | 1,29 | 0.0003 | 0.987 | 1,32 | 0.0015 | 0.970 | 1,32 | 0.2086 | 0.651 |
| N | Mi | 1,30 | 0.4979 | 0.486 | 1,29 | 2.4423 | 0.129 | 1,31 | 0.5371 | 0.469 |
| | Se | 1,30 | 0.3763 | 0.544 | 1,29 | 0.0196 | 0.890 | 1,31 | 0.4650 | 0.500 |
| | Mi*Se | 1,30 | 1.0931 | 0.304 | 1,29 | 0.2722 | 0.607 | 1,31 | 0.0083 | 0.928 |
| C/N ratio | Mi | 1,30 | 0.6306 | 0.433 | 1,31 | 6.1619 | **0.019** | 1,30 | 0.0025 | 0.960 |
| | Se | 1,30 | 0.7389 | 0.397 | 1,31 | 0.0376 | 0.847 | 1,30 | 0.0001 | 0.994 |
| | Mi*Se | 1,30 | 0.7231 | 0.402 | 1,31 | 0.9819 | 0.329 | 1,30 | 0.1185 | 0.733 |

§; Df: degree of freedom, (n,d): numerator and denominator of Df, F: F-statistics value, P: probability value.

**Table S7 ANOVA results for the LC-MS intensities of the selected metabolites in tomato leaves upon below- and aboveground herbivory.** LC-MS intensities of the selected metabolites were determined in leaves of tomato plants without herbivores (Control), infected with *Meloidogyne incognita* (Mi), or *Spodoptera exigua* (Se) alone, or double infected with both herbivores (MiSe). In double infected plants, infestation with *S. exigua* was performed at the nematode's galling stage. Samples were taken 24 hours after *S. exigua* feeding. Data were analyzed using a Two-way ANOVA linear model consisting of *M. incognita* (Mi), *S. exigua* (Se), and their interaction (Mi*Se) as model explanatory factors. The differences between the treatments were detected by Tukey's HSD test for multiple comparisons at $P \leq 0.05$. Statistically significant effects are indicated in bold.

| Mass to charge ratio (*m*/*z*), retention time (rt) in minutes | Predicted metabolites | Source of variation | Invasion | | | Galling | | | Reproduction | | |
|---|---|---|---|---|---|---|---|---|---|---|---|
| | | | $Df_{(n,d)}$ | F | P | $Df_{(n,d)}$ | F | P | $Df_{(n,d)}$ | F | P |
| *m*/*z* 203.053, rt 0.93 | Polyamine conjugated to a phenylpropanoid | Mi | 1,15 | 2.6628 | 0.124 | 1,11 | 71.523 | **<0.001** | 1,15 | 16.561 | **0.001** |
| | | Se | 1,15 | 12.1955 | **0.003** | 1,11 | 107.425 | **<0.001** | 1,15 | 25.902 | **0.000** |
| | | Mi*Se | 1,15 | 2.7301 | 0.119 | 1,11 | 10.194 | **0.009** | 1,15 | 21.363 | **0.000** |
| *m*/*z* 576.389, rt 5.74 | *α*-dehydrotomatine | Mi | 1,15 | 20.5819 | **0.000** | 1,14 | 0.8967 | 0.360 | 1,14 | 0.5416 | 0.474 |
| | | Se | 1,15 | 0.9470 | 0.346 | 1,14 | 1.6044 | 0.226 | 1,14 | 8.5433 | **0.011** |
| | | Mi*Se | 1,15 | 0.3747 | 0.550 | 1,14 | 1.0969 | 0.313 | 1,14 | 0.0774 | 0.785 |
| *m*/*z* 578.4056, rt 6.03 | *α*-tomatine | Mi | 1,13 | 7.5240 | **0.017** | 1,12 | 1.0117 | 0.334 | 1,14 | 0.0245 | 0.878 |
| | | Se | 1,13 | 0.1717 | 0.685 | 1,12 | 8.5875 | **0.013** | 1,14 | 6.1161 | **0.027** |
| | | Mi*Se | 1,13 | 4.2871 | **0.059** | 1,12 | 2.6976 | 0.126 | 1,14 | 0.0237 | 0.880 |
| *m*/*z* 188.0707, rt 3.44 | Unknown | Mi | 1,13 | 0.2646 | 0.616 | 1,13 | 1.0675 | 0.320 | 1,16 | 3.4628 | 0.0812 |
| | | Se | 1,13 | 12.2886 | **0.004** | 1,13 | 313.3833 | **<0.001** | 1,16 | 35.1683 | **<0.001** |
| | | Mi*Se | 1,13 | 0.7048 | 0.416 | 1,13 | 4.6465 | **0.050** | 1,16 | 3.6944 | 0.0726 |
| *m*/*z* 348.187 rt 4.5 | Unknown | Mi | 1,16 | 0.0216 | 0.885 | 1,13 | 15.731 | **0.007** | 1,15 | 1.3795 | 0.259 |
| | | Se | 1,16 | 18.1433 | **0.000** | 1,13 | 330.364 | **<0.001** | 1,15 | 16.3314 | **0.001** |
| | | Mi*Se | 1,16 | 0.0213 | 0.886 | 1,13 | 23.491 | **0.000** | 1,15 | 3.2609 | 0.091 |

§; Df: degree of freedom, (n,d): numerator and denominator of Df, F: F-statistics value, P: probability value.

## SUPPLEMENTARY FIGURES

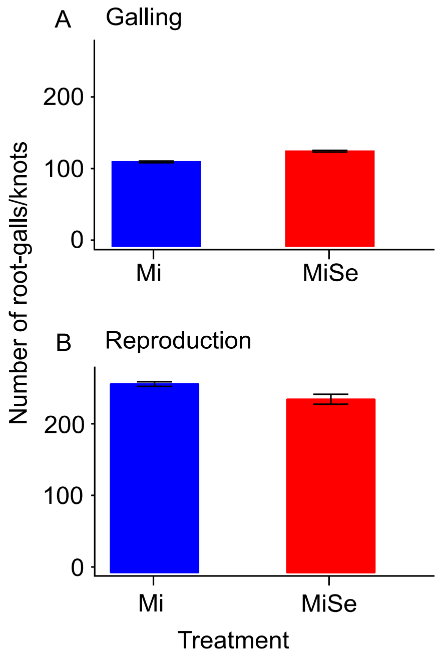


**Fig. S1 Number of *Meloidogyne incognita* galls/knots in tomato roots**. The average number of root galls/knots counted in tomato roots infected with *Meloidogyne incognita* alone (Mi) or double infected with *M. incognita* and *Spodoptera exigua* (MiSe). Roots were harvested at the nematode's galling (A) and reproduction (B) stages. Data are the mean ± standard error ($n$ = 10).

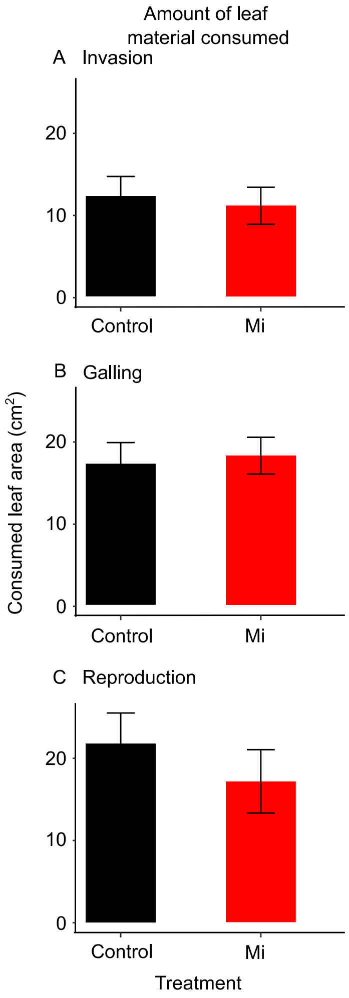


**Fig. S2 Amount of leaf material consumed by *Spodoptera exigua*.** The average feeding rate of *Spodoptera exigua* determined as leaf area in $cm^2$. Second-instar *S. exigua* larvae were added and allowed to feed for five days on tomato plants without root infection (Control) or infected with *Meloidogyne incognita* (Mi). The leaves were harvested at the nematode's invasion (A), galling (B), and reproduction (C) stages for the determination of leaf areas. Data are the mean ± standard error (*n* = 10).
